# Repeat HIV Testing at Voluntary Testing and Counseling Centers in Croatia: Successful HIV Prevention or Failure to Modify Risk Behaviors?

**DOI:** 10.1371/journal.pone.0093734

**Published:** 2014-04-04

**Authors:** Vlatka Matković Puljić, Mirjana Lana Kosanović Ličina, Marija Kavić, Tatjana Nemeth Blažić

**Affiliations:** 1 University Hospital for Infectious Diseases, Dr. Fran Mihaljević, Zagreb, Croatia; 2 Institute of Public Health Dr. Andrija Štampar, Zagreb, Croatia; 3 National Institute of Public Health, Zagreb, Croatia; Alberta Provincial Laboratory for Public Health/University of Alberta, Canada

## Abstract

HIV testing plays a critical role in preventing the spread of the virus and identifying infected individuals in need of care. Voluntary counseling and testing centers (VCTs) not only conduct testing but they also provide counseling. Since a proportion of people who test negative for HIV on their previous visit will return for retesting, the frequency of retesting and the characteristics of those who retest may provide insights into the efficacy of testing and counseling strategies. In this cross-sectional, retrospective study of 1,482 VCT clients in Croatia in 2010, 44.3% had been tested for HIV before. The rate of repeat HIV testing is lower in Croatia than in other countries. Men who have sex with men (MSM) clients, those with three or more sexual partners in the last 12 months, consistent condom users with steady partners, and intravenous drug users were more likely to be repeat testers. This finding suggests that clients presenting for repeat HIV testing are those who self-identify as being at a higher risk of infection. Our data showed that testing positive for HIV was not associated with repeat testing. However, the effects of repeat testing on HIV epidemiology needs to be explored.

## Introduction

Voluntary counseling and testing centers (VCTs) are the front-line actors in HIV prevention strategies in most countries [1]. VCTs allow individuals to learn their human immunodeficiency virus (HIV) serostatus in a confidential environment, and they provide pre- and post-test counseling about HIV, risk factors, and prevention strategies. Clients can discuss their personal risk profile (sexuality, drug use) and receive guidance on how to change their and perhaps their partners’ risk behaviors [2]. In this way, VCTs play an important dual role: first, in preventing HIV infection by encouraging behavioral change; and secondly, in enhancing early presentation to care by serving individuals who test HIV-positive as an entry point and/or referral to care and treatment.

A proportion of clients who have already tested negative for HIV return to VCTs to be tested again. The reasons for this are varied: because of their lifestyle or other risk factors; repeat testers may perceive themselves as more vulnerable to acquiring HIV and therefore feel the need for continuous vigilance; they may be confused about risk and decide to “err on the side of caution”; or they may feel the need to reassure their uninfected partners that they are virus-free [3], [4]. Repeat testers present numerous differences from those with no prior history of HIV testing. According to a cohort study in the United States, they are more likely to be men who have sex with men (MSM), to have a history of drug use [5], to report recent risk behaviors, and to test positive for HIV [6]. Repeat testers from Thailand are more likely to be male sex workers, older, and employed, to live away from the family, and to have an insertive anal sex role [7].

Studies in the United States indicate that the rate of repeat HIV testing has increased considerably during the 1990s and 2000s [5]. For instance, 31% of all tests performed in San Francisco in 1992 involved repeat testers, while 65% of all tests performed in seven American cities in 2002 involved repeat testers [5], [6]. This prompts the question of why people retest [8]: is it because they continue to engage in high-risk behaviors, or is it because their initial negative testing reinforces their low-risk behavior and they want to reassure themselves that they remain uninfected?

Understanding factors related to repeat testing may help public health practitioners and policy makers assess the efficacy of HIV testing and counseling strategies. It may also help counselors tailor their pre- and post-test interventions and referral practices according to client profiles.

To gain further insight into the characteristics of repeat HIV testers, we performed a cross-sectional, retrospective study of clients at the two largest VCTs in Croatia. Our findings may help improve HIV testing and prevention policies in the country and the region.

## Methods

This study included all individuals at least 16 years old who underwent an HIV test in 2010 at one of the two study sites and who provided data on HIV testing history.

### Description of Study Sites and General Practices

Data on HIV testing in 2010 was collected from the two largest VCTs in Croatia, both of which are located in the capital city of Zagreb: the VCT at the National Institute of Public Health (NIPH) and the VCT at the University Hospital for Infectious Diseases “Dr. Fran Mihaljević” (UHID). Every year, these two VCTs conduct approximately 1,500 HIV tests, accounting for 75% of all anonymous tests conducted in the country.

Both VCTs in this study provide free, anonymous and confidential HIV testing based on random coding, together with confidential pre- and post-test counseling. A total of 8 counselors were working at the two study sites in 2010 (6 at NIPH, 2 at UHID). All counselors were university-educated in psychological or medical sciences and had been trained as “HIV counselors” in a 3-day course conducted by the National Institute of Public Health using a curriculum designed according to the Centers for Disease Control and Prevention (CDC), the Joint United Nations Programme on HIV/AIDS (UNAIDS), and the World Health Organization (WHO) guidelines [9]–[11]. Counseling sessions were client-centered and delivered according to abovementioned guidelines. Pre-test counseling sessions were structured as interviews aimed at determining individual HIV risk, providing information about the HIV test and HIV transmission, explaining ways to reduce personal risk, the importance of learning the test results, and clarifying the meaning of HIV test results. HIV test results were delivered as part of a post-test session in which the results were discussed, personal risk reduction strategies were further explored, and referrals made when appropriate [12], [13]. Free condoms and brochures on HIV and sexually transmitted infections (STIs) were provided at pre- and post-test sessions.

At the time of HIV testing, clients were also offered the possibility of free testing for hepatitis B virus (HBV) and hepatitis C virus (HCV). Results of the HIV, HBV, and HCV tests were available within seven days after testing. VCTs in Croatia require clients to return for test results in person, but exceptions are allowed in special circumstances, such as when the client lives in a remote part of the country, is travelling or is moving to another country. In these cases, results are communicated by phone.

### Questionnaire and Blood Collection

Data for this study were collected retrospectively from responses to a questionnaire filled out by VCT counselors during the pre-test interview with clients. This questionnaire, used in all VCTs in the country, was designed by the Croatian National Institute of Public Health [14] according to guidelines of CDC, WHO, and of UNAIDS as part of the project “Scaling up the HIV/AIDS response in Croatia” in 2004 to 2006, funded by The Global Fund to Fight AIDS, Tuberculosis and Malaria [15].

The questionnaire included items on socio-demographic data, sexual risk behavior, history of STIs, and history of HIV testing. We compared these items between clients who were repeat HIV testers and those who were first-time testers. Repeat HIV testers were those who self-reported having previously tested negative for HIV and who were now returning for another HIV test.

Clients were required to answer only questions related to essential socio-demographic data, and they were free to refuse to answer or discuss the additional, optional questions with the counselor. Clients who did not provide any responses to the optional questions were excluded from the study (n = 50). Those who provided answers to at least some of the optional questions were included (n = 1482). This gave rise to gaps in the data set, such that frequencies for some variables did not add up to the total number of clients. Blood was drawn from all clients wishing to be tested for HIV and/or HBV/HCV, regardless of their willingness to provide answers to the optional questions in the pre-test counseling session.

### Variables

Data were collected on the following client characteristics: socio-demographics (age, sex, education level, residence, sexual orientation, marital status), HIV testing history, number of sexual partners in the last 12 months, condom use with steady and casual partners, principal reason for not using condoms, intravenous drug use, and STI history.

Education level was treated on the questionnaire as a categorical variable with a value from 1 (no qualification) to 5 (doctoral degree). During data analysis, the small number of clients with no education or only primary school qualifications prompted us to dichotomize the variable into 1 (high school or less) and 2 (university or higher). Similarly, consistent condom use with steady and casual partners (as defined by the client) was reported on the questionnaire using a 4-point scale from “never” to “always”. During data analysis, this variable was dichotomized into “consistent condom use” (always uses condoms) and “inconsistent condom use” (never, occasionally or rarely uses condoms).

### Statistical Analysis

Calculations and statistical tests were performed using R package version 2.15.1 (http://www.r-project.org/) including libraries *epitools* and *prettyR*. We reported results for continuous variables using the median and interquartile range (IQR). Frequencies were compared using the chi-squared test or Fisher’s exact test when observed numbers were small. Odds ratios (ORs) and 95% confidence intervals (CIs) were used to describe the relationships among HIV testing history as an outcome variable; and socio-demographic characteristics and risk behaviors as predictors. Median-unbiased estimation was used to calculate OR.

We also calculated OR and CI of repeat testing in non-respondents for all variables with more than 10% missing data.

A generalized linear model was used to develop a multivariable binary logistic regression model. The regression model was calculated using the ‘Enter’ method in which all variables in a block are entered in a single step. Variables were assessed for inclusion based on their statistical significance in bivariable analysis (with a significance threshold of p<0.1), missing data, collinearity problems, and on their expected importance based on public health policy and practice. All variables were included in the analysis in one block. Although statistically significant, we excluded from the model two variables with more than 50% missing data (consistent condom use with casual partners, and condom use at most recent sexual intercourse with a casual partner). The variable *sex* was also omitted due to high collinearity with the sexual orientation variable.

### Ethics Statement

The study protocol and procedure for obtaining informed consent were approved by the Ethics Committees of the National Institute of Public Health (project ID 0001-53311-12, 18 September 2012) and of the University Hospital for Infectious Diseases “Dr. Fran Mihaljević” (project ID VCT 2008–2010, 12 July 2010).

All clients gave verbal informed consent to voluntary, anonymous, and confidential HIV testing. Before giving consent, clients were informed verbally about the HIV testing and counseling that would be performed, the availability of opt-in HBV/HCV testing, and the type of data that would be collected during the counseling sessions. They also received verbal explanations of HIV infection and the meaning of positive and negative test results, after which clients were offered the opportunity to ask questions and decline any part of the testing and counseling process. The same information and consent procedures were used for minors as for adults, consistent with CDC guidelines on obtaining verbal consent for HIV testing for adults and adolescents [16]. Since this study involved retrospective analysis of routinely collected anonymous data in VCTs, we did not develop a consent form specifically for this study, nor did we seek informed consent from parents or guardians of adolescents. If a patient declined to provide data, this decision was documented on the counseling form with the note “Does not wish to share information” and the patient was excluded from the study. Ethics Committees of National Institute of Public Health and University Hospital for Infectious Diseases “Dr. Fran Mihaljević” approved the consent procedure.

## Results

### Socio-demographic Characteristics of Study Participants

In 2010, 1,482 clients sought HIV testing at UHID (970, 65.5%) and NIPH (512, 34.5%). A total of 657 clients (44.3%) reported having been tested for HIV previously, while the remaining 55% were being tested for the first time. The study sample comprised 977 (66.6%) men, 378 (25.8%) of whom were classified as MSM. Most clients were from urban areas (90.5%), and 844 (57.3%) had a high school education or less. Just over half of the participants reported not being in a relationship (n = 724, 51.2%), similar to the number who reported being married or in a stable relationship (n = 690, 48.8%). Half the participants (769, 51.9%) opted-in for HBV/HCV testing. A small percentage of participants did not return for test results (n = 94, 6.9%).

Table 1 presents the demographic characteristics and risk behavior patterns for repeat HIV testers and first-time testers.

**Table 1:** Socio-demographic and risk behavior characteristics among 1,482 first-time and repeat testing clients at voluntary HIV testing and counseling centers in Zagreb, Croatia.

Variable	Value	Respondents with the givenvalue, n (%)	Repeat testers,n (%),	First-time testers, n (%),
			(N = 657)	(N = 825)
Residence				
	Rural area	140 (9.5)	45 (32.1)	95 (67.9)
	Urban area	1331 (90.5)	608 (45.7)	723 (54.3)
Sex				
	Female	489 (33.2)	163 (33.3)	326 (66.7)
	Male	985 (66.8)	490 (49.7)	495 (50.3)
Age (y)				
	16–25	426 (28.8)	136 (31.9)	290 (68.1)
	26–30	414 (28.0)	182 (44.0)	232 (56.0)
	31–40	441 (29.8)	240 (54.4)	201 (45.6)
	≥41	199 (13.4)	99 (49.7)	100 (50.3)
Education level				
	High-school or lower	844 (57.3)	329 (39.0)	515 (61.0)
	University or higher	630 (42.7)	323 (51.3)	307 (48.7)
Relationship status				
	Married or in stable relationship	690 (48.8)	284 (41.2)	406 (58.8)
	Not in relationship	724 (51.2)	333 (46.0)	391 (54.0)
Sexual orientation				
	Heterosexual men	599 (40.9)	221 (36.9)	378 (63.1)
	MSM	378 (25.8)	265 (70.1)	113 (29.9)
	Heterosexual women	489 (33.4)	163 (33.3)	326 (66.6)
Number of sexual partners in last 12 months				
	<3	638 (55.5)	227 (35.6)	411 (64.4)
	≥3	512 (44.5)	268 (52.3)	244 (47.7)
Self-reported STI history*				
	Never	986 (82.6)	411 (41.7)	575 (58.3)
	Ever	207 (17.4)	98 (47.3)	109 (52.7)
Consistent condom use with steady partner				
	No	728 (86.3)	283 (38.9)	445 (61.1)
	Yes	116 (13.7)	69 (59.5)	47 (40.5)
Consistent condom use with casual partner				
	No	467 (69.1)	192 (41.1)	275 (58.9)
	Yes	209 (30.9)	114 (54.5)	95 (45.5)
Condom use at last sexual intercourse				
	No	505 (55.0)	196 (38.8)	309 (61.2)
	Yes	414 (45.0)	183 (44.2)	231 (55.8)
Condom use at last sexual intercourse with casual partner				
	No	424 (66.1)	169 (39.9)	255 (60.1)
	Yes	217 (33.9)	109 (50.2)	108 (49.8)
Intravenous drug user				
	No	1027 (98.0)	427 (41.6)	600 (58.4)
	Yes	21 (2.0)	14 (66.7)	7 (33.3)
HIV status				
	Positive	29 (2.0)	12 (41.4)	17 (58.6)
	Negative	1453 (98.0)	645 (44.4)	808 (55.6)
HBV status				
	Positive	1 (0.1)	0 (0.0)	1 (100.0)
	Negative	768 (99.9)	349 (45.4)	419 (54.6)
HCV status				
	Positive	8 (1.0)	3 (37.5)	5 (62.5)
	Negative	761 (99.0)	347 (45.6)	414 (54.4)
Returned for test results				
	No	94 (6.9)	35 (37.2)	59 (62.8)
	Yes	1274 (93.1)	565 (44.3)	709 (55.7)

*STI, sexually transmitted infections.

Frequencies may not add up to the total number of respondents (N = 1482) because of missing data.

### HIV Seroprevalence

Of all 1,482 clients, 29 (2.0%) tested HIV-positive. Of 769 clients tested for HBV/HCV, 8 (1.0%) were HCV-positive, and 1 (0.1%) was HBV-positive. None of the HIV positive people were found to be HBV or HCV co-infected. Just over half of those testing positive for HIV were being tested for the first time (n = 17, 58.6%), corresponding to 2.1% of all 825 clients being tested for the first time. Among 657 repeat testers, 12 (1.8%) tested HIV positive. Among the 378 clients who self-reported as MSM, 22 (5.8%) tested positive for HIV; this number was split evenly between 11 first-time testers (9.7%) and 11 repeat testers (4.2%). Hence, those MSM who test positive were more likely to be first-time testers (OR 2.48, 95% CI 1.02 to 6.03, P = 0.033). Among heterosexual men and women, first-time and repeat testers showed similar rates of testing HIV positive. Of the first-time testers, five (1.3%) heterosexual men and one (0.3%) woman seroconverted, while one (0.5%) man and no women among repeat testers tested positive.

### Factors Associated with Repeat HIV Testing

Table 2 explores associations among client demographics, medical and behavioral characteristics and repeat HIV testing. The following factors were found to be associated with repeat testing: living in urban areas, male sex, being MSM, age above 25, university education, having three or more sexual partners in the last 12 months, consistent condom use with steady and casual partner, condom use at last casual sex, and being IDU.

**Table 2:** Bivariable assessment of socio-demographic and risk behavior characteristics associated with repeat HIV testing.

Variable	Value	Respondents on the variable, n	OR (95% CI) of repeat testing	P
Residence	Urban (vs rural)	1471	1.77 (1.22–2.58)	0.002
Sex	Male (vs Female)	1474	1.97 (1.58–2.48)	<0.001
Age (y)	26–30 (vs 16–25)	1480	1.67 (1.26–2.21)	<0.001
	31–40 (vs 16–25)		2.54 (1.93–3.35)	<0.001
	≥41 (vs 16–25)		2.11 (1.49–2.97)	<0.001
Education	University or higher (vs High-school or lower)	1474	1.64 (1.33–2.02)	<0.001
Relationship status	Not in relationship (vs Married or in stable relationship)	1414	1.21 (0.98–1.50)	0.067
Sexual orientation	MSM (vs Heterosexual men)	1466	4.01 (3.04–5.28)	<0.001
	Heterosexual women (vs Heterosexual men)		0.85 (0.66–1.09)	0.221
Number of sexual partners in last 12 months	≥3 (vs <3)	1150	1.98 (1.56–2.52)	<0.001
History of self-reported STI*	Ever (vs Never)	1193	1.25 (0.93–1.69)	0.134
Consistent condom use with steadypartner	Yes (vs No)	844	2.30 (1.54–3.45)	<0.001
Consistent condom use with casualpartner	Yes (vs No)	676	1.71 (1.23–2.38)	0.001
Condom use at last sexual intercourse	Yes (vs No)	919	1.24 (0.95–1.62)	0.099
Condom use at last sexual intercoursewith casual partner	Yes (vs No)	641	1.52 (1.09–2.11)	0.012
Intravenous drug user	Yes (vs No)	1048	2.77 (1.13–7.48)	0.021
HIV status	Negative (vs Positive)	1482	1.12 (0.53–2.45)	0.746
HCV status	Negative (vs Positive)	769	1.36 (0.33–7.14)	0.647
Returned for test results	Yes (vs No)	1368	1.34 (0.87–2.08)	0.179

*STI, sexually transmitted infections.

Repeat testers reported a higher number of sexual partners in the last 12 months (3, IQR 1–5) than did first-time testers (2, IQR 1–3; P<0.001). Furthermore, the number of sexual partners was significantly higher for MSM (4, IQR 2–7) than for heterosexual men (2, IQR 1–4) and women (1.5, IQR 1–2; P<0.001). Clients who tested HIV positive in our study had more sexual partners in the last 12 months (3, IQR 1–6) than did those who tested negative (2, IQR 1–4; P = 0.042).

Moreover, clients with a history of gonorrhea (OR 3.27, 95% CI 1.07 to 12.41; P = 0.053) or syphilis (OR 9.56, 95% CI 2.65 to 66.15; P<0.001) were more likely to be repeat testers than those who did not report having those STIs.

Consistent condom users with casual partners and condom use during the most recent sexual intercourse with a casual partner were significantly associated with repeat HIV testing. However, a multivariable analysis confirmed that consistent condom users with steady partners were more likely to be repeat testers (OR 2.31, 95% CI 1.26 to 4.26; P<0.007). Among the 704 clients who gave a reason for not using condoms, 567 (80.5%) cited trust in their partner, 83 (11.8%) said that they did not like wearing one, and 29 (4.1%) indicated that a condom was not available at the time of intercourse.

Table 3 presents factors predictive of repeat HIV testing. A total of 548 clients (36.9%) were included in the final model. The regression model was statistically significant (χ2 = 88.307, df = 12, p<0.001) with a 70.3% chance to correctly predict the outcome variable (repeat HIV testing). Sexual orientation (MSM clients), number of sexual partners in the last 12 months, consistent condom use with steady partner and intravenous drug use were statistically significant predictors in this model.

**Table 3:** Multivariable assessment of socio-demographic and risk behavior characteristics associated with repeat HIV testing.

Variable	Value	Respondents with the given value, n	OR (95% CI) of repeat testing	P
		(N = 548)		
Residence	Urban (vs rural)	506	1.91 (0.85–4.29)	0.119
Age (y)				
	26–30 (16–25)	180	1.54 (0.91–2.61)	0.105
	31–40 (16–25)	153	2.04 (1.15–3.61)	0,014
	≥41 (16–25)	58	1.58 (0.75–3.31)	0.231
Education	University or higher (vs High-school or lower)	254	1.30 (0.85–1.99)	0.225
Relationship status	Not in relationship (vs Married or in stable relationship)	224	1.32 (0.89–1.95)	0.167
Sexual orientation				
	MSM (vs Heterosexual men)	140	3.60 (2.24–5.79)	<0,001
	Heterosexual women (vs Heterosexual men)	175	1.24 (0.78–1.96)	0.364
Number of sexual partners in last 12 months	≥3 (vs <3)	242	1.57 (1.05–2.34)	0,026
Consistent condom use with steadypartner	Yes (vs No)	66	2.31 (1.26–4.26)	0,007
Condom use at last sexual intercourse	Yes (vs No)	240	0.98 (0.66–1.50)	0.992
Intravenous drug user	Yes (vs No)	5	9.43 (1.00–90.9)	0,05
Constant				0.178

Note: Large number of missing data (see Table 4).

The association of repeat testing with characteristics of non-respondents and respondents are presented in Table 4. We found that those who did not respond to the variables about history of self-reported STI, consistent condom use with steady partner, condom use at last sexual intercourse with casual partner, and intravenous drug use were more likely to be repeat testers than those who did respond.

**Table 4:** Analysis of the characteristics of non-respondents on variables with >10% missing data.

	Repeat testers on the given variable			
Variable	Non-respondents, n (%)	Respondents, n (%)	OR (95% CI) of repeat testing in non-respondents (vs respondents)	P
Number of sexual partners in last 12 months	162 (48.8)	495 (43.0)	1.26 (0.99–1.61)	0.063
History of self-reported STI*	148 (51.2)	509 (42.6)	1.41 (1.09–1.83)	0.009
Consistent condom use with steady partner	305 (47.8)	352 (41.7)	1.28 (1.04–1.58)	0.019
Consistent condom use with casual partner	351 (43.5)	306 (45.1)	0.93 (0.76–1.15)	0.507
Condom use at last sexual intercourse	278 (49.4)	379 (41.2)	1.07 (0.87–1.32)	0.515
Condom use at last sexual intercourse withcasual partner	379 (45.1)	278 (43.4)	1.39 (1.13–1.72)	0.002
Intravenous drug user	216 (49.8)	441 (42.1)	1.36 (1.09–1.71)	0.007
HBV status	308 (43.2)	349 (45.4)	0.92 (0.75–1.12)	0.397
HCV status	307 (43.1)	350 (45.5)	0.91 (0.74–1.11)	0.342

*STI, sexually transmitted infections.

## Discussion

We retrospectively analyzed factors associated with repeat HIV testing in 2010 at two VCTs covering approximately 75% of all anonymous HIV tests in Croatia. Our study population comprised predominantly younger heterosexual adults from urban areas with a high school education. Less than half of the study participants were repeat testers.

Overall 44% of our study population were repeat testers, which is lower than the >50% reported in similar studies in several countries [4], [7], [17]–[20] and substantially lower than the 63% reported among patients in an STI clinic and 70% in an HIV screening program in the US [17], [19]. This lower frequency of repeat testing may reflect the fact that the HIV epidemic in Croatia is at a low level; prevalence in general population <1% and prevalence in each most at-risk population <5% [21]. We found an HIV prevalence of 2% among VCT clients and a slightly lower prevalence of 1.8% among repeat testers. National data with which to compare our study results are limited. Between 2001 and 2005, the rate of HIV-positive tests in VCTs and clinics across the country was below 1.5% [15]. In 2010, 68 new cases of HIV were reported. Since the VCT system began operating in Croatia under the auspices of the Global Fund program in 2004, 30–40% of new HIV infections are diagnosed in these centers annually.

Two-thirds of MSM in our study were repeat HIV testers, and they were four times more likely to undergo repeat HIV testing than heterosexual men. The multivariable model confirms the association between MSM and frequency of repeat testing. The frequency of 70.1% found in our study is higher than the corresponding values found in respondent driven sampling (RDS) surveys of HIV seroprevalence among MSM in Croatia, the Netherlands, and Thailand [7], [22]–[23]. A French and a Swiss study among MSM both reported that a large majority of the respondents had been tested at least once in their lives; and this proportion increased over time [4], [20]. Similar studies conducted in Thailand, UK, and US have shown MSM to be an independent predictor of repeat HIV testing [7], [18], [24]. These high rates of repeat HIV testing among MSM suggest that they may see themselves as a group at high risk of acquiring HIV. Future research should examine the effects of this behavior on HIV epidemiology.

We found the prevalence of HIV-positive results to be more than twice as high among first-time MSM testers (9.7%) as among MSM retesters (4.2%). These results are similar to those reported in a same-day HIV testing clinic in the UK and the Netherlands, but much higher than those reported for MSM testing at VCTs in Switzerland [18], [20], [23]. In Croatia, surveys of HIV seroprevalence among MSM indicate a prevalence of 4.5% in 2006 and 2.8% in 2010 [25], suggesting that the HIV epidemic may not have advanced in this population over the last 5 years.

Recent studies suggest that retesting may be a powerful public health strategy for preventing and detecting HIV infection as well as for initiating HIV treatment earlier. The fact that an increasing proportion of individuals in the US have been tested for HIV has been suggested as a reason why fewer infected individuals are diagnosed late [26]. Individuals diagnosed early may be less likely to transmit the virus to others, not only because treatment has reduced their infectivity but also because they have modified their risk behaviors [27]. In a study in Croatia, Begovac et al. showed that HIV-infected heterosexuals seek medical treatment at a later stage of infection than do MSM because heterosexuals test for HIV less often than MSM [28]. This may be because heterosexuals perceive themselves to be at lower risk of infection. Future research should examine whether infected individuals who present early for treatment also tend to be repeat HIV testers.

Current policy in Croatia recommends testing for individuals engaging in high-risk behaviors. This policy may be ineffective in the case of individuals who fail to self-identify risk behaviors or who nevertheless perceive themselves to be at lower risk of acquiring HIV. Such individuals may be more likely to be unaware HIV carriers who transmit infection and present late to treatment, resulting in a worse prognosis [28].

Raising HIV risk awareness among Croatians through HIV testing is especially needed because of low rates of condom use when compared to other countries. In our study, rates of consistent condom use with a steady partner were 9% for first-time testers and 19% for repeat testers. The rates were somewhat higher when sex involved casual partners: 25% for first-time testers and 37% for repeat testers. By comparison, studies in the US and UK showed rates of condom use with any partner to be, respectively, 41% and 44% for first-time testers and 47% and 49% for repeat testers [17], [18]. Our low rates of condom use are consistent with an earlier study of Croatian young adults, which found that one-fifth used condoms consistently over the past 12 months, and slightly over half used condoms during their last sexual intercourse [29].

Only 21 (2.0%) of our VCT clients reported a history of intravenous drug use, and 57% of them were repeat testers. None of the intravenous drug users in our study was positive for HIV or HBV, while 3 (21%) were HCV-positive. HIV prevalence among intravenous drug users in Croatia has remained stable around 0.5% over the past decade [30], [31], which may reflect in part the efficacy of drug user registration and intervention programs.

There are some limitations to this cross-sectional and retrospective study. First, the cross-sectional nature of the study limits the interpretation of causality. Secondly, data were collected from a convenience sample at two VCTs in one city, raising the possibility of selection bias, such that the characteristics and risk behaviors of our study population may differ from those of other parts of the country [32]. Nevertheless, the two sites in this study accounted for 75% of all VCT HIV testing in Croatia in 2010. Thirdly, substantial self-selection bias can ensue because non-respondents are somewhat different from respondents (Table 4). However, characteristics of respondents and non-respondents who were repeat testers were comparable. The main reasons for non-response may be the predisposing behavioral factors, the length and/or sensitive nature of the HIV counseling and testing process. However, we did not collect information on the reasons for non-responding. Fourthly, since our data were collected through interviews, interviewer bias and recall bias of clients cannot be excluded. To reduce interview bias, only well trained counselors participated in our study. The fact that HIV testing was anonymous probably reduced pressure on clients to give socially desirable answers. Future work should examine whether risk behaviors and attitudes or perceptions of HIV risk change significantly between visits to the VCT.

## Supporting Information

Questionnaire S1 File Questionnaire S1 is an English translation of the questionnaire used in the counseling process and filled out by VCT counselors during the pre-test interview with clients.
(DOC)
