# Supplementary material for: Repeat HIV Testing at Voluntary Testing and Counseling Centers in Croatia: Successful HIV Prevention or Failure to Modify Risk Behaviors?
Source: PLoS One. 2014 Apr 4;9(4):e93734. doi: 10.1371/journal.pone.0093734 (PMC3976312; doi:10.1371/journal.pone.0093734)
Supplement: Questionnaire S1 — File Questionnaire S1 is an English translation of the questionnaire used in the counseling process and filled out by VCT counselors during the pre-test interview with clients. (DOC) [file pone.0093734.s001.doc]

***Mandatory part***

To be filled out by counselor

Voluntary and Counseling Testing Center (VCT) location* _________________________________________________________________

| **Code number** | **Date** | **Counselor name and surname** |
| --- | --- | --- |
|  |  |  |

***VCT location**

**1-** University Hospital for Infectious Diseases „Dr. Fran Mihaljević“ (UHID)

**2-** National Institute of Public Health (NIPH)

**Place of residence**

country

small town

town

S**ex**

male

female

**Year of birth**

____yyyy

**Education**

elementary school

secondary school qualification

university qualifications

*Risk behaviour*

**Sexual contact without condom: anal and/or vaginal**

No

Yes

**Injecting drug user IDU ever**

No

Yes

**Client’s most recent risk behavior activities**

<= 3 months window period

> 3 months

**Client's risk behavior within last 6 months**

one time

multiple times

***Optional part to be filled out only if client agrees***

***Client does not wish to share information***

**Marital status**

married

single

partnered

divorced

separated

widowed

**Sexual orientation**

heterosexual

homosexual

bisexual

**Number of sexual partners in last 12 months**

_____ exact number

**Condom use with steady partner**

always

most of the time/often

sometimes

never

**Condom use with casual partner**

always

most of the time/often

sometimes

never

**Reason for not using condoms**

do not like sex with condom

trust in partner

too expensive

embarrassed when buying condoms

difficult to use

not available

embarrassed to ask a partner to use condom

**Condom use at last sexual intercourse with casual partner?**

No

Yes

**Condom use at last sexual intercourse?**

No

Yes

**Previously tested on HIV?**

never tested on HIV

tested on HIV sometimes in the past

**Has client ever been diagnosed with any STI?**

No

Yes

**Has client ever been diagnosed with Chlamydia?**

No

Yes

**Has client ever been diagnosed with Gonorrhea?**

No

Yes

**Has client ever been diagnosed with HPV?**

No

Yes

**Has client ever been diagnosed with HSV?**

No

Yes

**Has client ever been diagnosed with Syphilis?**

No

Yes

**Has client injected drugs intravenously within last month?**

No

Yes

**If answer to last question is yes, has client shared injecting equipment?**

No

Yes

*History of testing*

**HBsAg tested**

No

Yes

**antiHCV tested**

No

Yes

**HBsAg result**

negative

positive

**antiHCV result**

negative

positive

**antiHIV result**

negative

positive

**Returned for HIV testing results**

No

Yes
